# Why does an obligate autogamous orchid produce insect attractants in nectar? – a case study on *Epipactis albensis* (Orchidaceae)

**DOI:** 10.1186/s12870-022-03563-3

**Published:** 2022-04-13

**Authors:** Anna Jakubska-Busse, Izabela Czeluśniak, Michał J. Kobyłka, Marek Hojniak

**Affiliations:** 1 University of Wroclaw, Faculty of Biological Sciences, Department of Botany, 50-328 Wroclaw, Poland; 2 University of Wroclaw, Faculty of Chemistry, 50-353 Wroclaw, Poland

**Keywords:** *Epipactis*, Orchids, Autogamy, Flower visitor attraction, Floral scent, Floral volatiles, Nectar chemical composition, GC–MS

## Abstract

**Background:**

The flowers of some species of orchids produce nectar as a reward for pollination, the process of transferring pollen from flower to flower. *Epipactis albensis* is an obligatory autogamous species, does not require the presence of insects for pollination, nevertheless, it has not lost the ability to produce nectar, the chemical composition of which we examined by gas chromatography-mass spectrometry (GC–MS) method for identification of potential insect attractants.

**Results:**

During five years of field research, we did not observe any true pollinating insects visiting the flowers of this species, only accidental insects as ants and aphids. As a result of our studies, we find that this self-pollinating orchid produces in nectar *inter alia* aliphatic saturated and unsaturated aldehydes such as nonanal (pelargonal) and 2-pentenal as well as aromatic ones (i.e., syringaldehyde, hyacinthin). The nectar is low in alkenes, which may explain the absence of pollinating insects. Moreover, vanillin and eugenol derivatives, well-known as important scent compounds were also identified, but the list of chemical compounds is much poorer compared with a closely related species, insect-pollinating *E. helleborine*.

**Conclusion:**

Autogamy is a reproductive mechanism employed by many flowering plants, including the orchid genus *Epipactis*, as an adaptation to growing in habitats where pollinating insects are rarely observed due to the lack of nectar-producing plants they feed on. The production of numerous chemical attractants by self-pollinated *E. albensis* confirms the evolutionary secondary process, i.e., transition from ancestral insect-pollinating species to obligatory autogamous.

## Background

One of the largest families of plants, Orchidaceae, is mainly known for producing sophisticated, colorful, an elegant and fragrant flowers and complicated system of pollination mechanisms. On one side, these flowering plants have adapted the shape of their flowers to attract pollinating animals. On the other hand, they attract pollinators by producing chemicals that act as the pheromones and also, they are capable of producing nectar rich in sugars, amino acids, as well as minerals [1–3]. In general, orchids also offer their pollinators a variety of floral food-rewards, such as oil and edible trichomes, with many more producing non-food rewards, such as fragrances, waxes and resins [2]. In these plants nectar is the most common floral food-reward, but they have also developed strategies for using insects without rewarding them. A floral and/or sexual deception are common strategy in orchids’. Apart from manipulating the behavior of pollinators, nectar can also have another function, i.e., inhibit the growth of microorganisms, protect/prevent against nectar robbers, herbivores and pathogens [3–8].

Floral nectar is the primary source of carbohydrates and constitutes the major energy source for visitors and defensive mutualists [3, 8]. The main ingredients in nectar are a watery solution of sugars, i.e., sucrose, glucose and fructose, and rarely of other carbohydrates e.g., mannose, xylose, and maltose, but it also contains traces of proteins, salts and essential oils [1, 3, 7]. Besides sugars, nectar contains a wide range of different organic acids, vitamins, alkaloids, phenolics, terpenoids, lipids, metal ions and hormones and secondary compounds to attract pollinators [1, 3, 7, 8].

Floral nectar is synthesized and produced in glands called nectaries and collected by different group of pollinators such as fruit-eating bats, hummingbirds, sunbirds, and insects and other animals [9]. In orchids nectaries are usually located at the base of the lip, in the concave basal part of labellum called hypochile or at the base of the flower alongside the ovary and on the side lobes, or along the central groove of the labellum, as well as in long spurs [7, 8, 10, 11].

Characteristic feature of orchid morphology is the grouping of the entire production of pollen grains of every flower into two or more discrete masses, termed pollinia, with two or more of these combined into a structure called the pollinarium [12]. True pollinia and pollinaria occur only in two subfamilies of the Orchidaceae, i.e., the Orchidoideae and the Epidendroideae [12], with the genus *Epipactis* belonging to the latter. Pollinaria differ in the degree of cohesion of pollen in the pollinium, which may be soft, sectile (comprised of sub-units known as massulae) or hard [13]. A single hard pollinium may contain more than a million pollen grains, yet pollen: ovule ratios in orchids are several orders of magnitude lower than in plants with powdery pollen due to the lack of wastage during transport to the stigma.

Pollinium is connected to the viscidium by the stalk which is composed of a caudicula (originated from the sporogenous tissue) and a stipe, derived from vegetative tissue, or be lacking altogether [13]. The viscidium is a sticky pad formed by the breakdown of stigmatic cells and which composition remains unknown. The sticky glue of the viscidium enables pollination as just after the contact to the body of the pollinators it dries out ensuring fixed adherence of pollinia to the pollinator;

The genus *Epipactis*, following the modern taxonomical concept includes 60–80 species [14, 15], both allogamous and autogamous taxa with considerable varietion in their floral morphology and complicated breeding system [16, 17]. Allogamous *Epipactis* species have a well-developed rostellum, a strict a single organ, formed by the modification of the dorsal stigma and the pistil [18], and produce the viscidium. In self-pollinating *Epipactis* species the latter organ disappears [19].

Species of the genus *Epipactis* offer visiting animals the superficial nectar on a lip, the nectary is placed in the hypochile [10]. In addition, the whole hypochile and also the knobs of epichile secrete nectar [10]. The scent possibly comes from the nectar aromatic compounds and is secreted through the epichile tissue and the osmophores [6, 20, 21].

*Epipactis albensis* Nováková et Rydlo is an autogamous species derived from the *E. helleborine* aggregate and it was formally described as a separate taxon in 1978 based on plants found in the flood plain forests adjoining the river Elbe in Central Bohemia [22]. Since that time, numerous localities of this species were reported from the Czech Republic [23], Slovakia [24], Romania, Austria, France, Germany, Poland [19, 25, 26] and Ukraine [27]. Two varieties (intraspecific taxa) are distinguished within the species, i.e., *Epipactis albensis* var. *albensis* and *E. alb*ensis var. *fibri* (Scappat. & Robatsch) P. Delforge [28]. Some researchers, based on morphological characters, distinguish a separate subspecies *Epipactis albensis* subsp. *lusatia* Hennigs [29].

*Epipactis albensis* is an obligate autogamous taxon [26], which means that pollen is transferred to the stigma of the same flower, but without the involvement of pollinating insects. The gynostemium of this species does not produce a viscidium, its rostellum is non-functional, a cavity in the upper part of the column that contains the anthers, called clinandrium, is reduced, anther sessile and pollinia are powdery [15, 30], therefore, theoretically it is not suitable for pollination by visiting insects. Interestingly, our observations showed that inflorescences of *E. albensis* are occasionally visited by insects. Because the species does not produce the viscidium, even when the flowers are penetrated by insects, the pollinia do not attach to their bodies and therefore remain inside the flowers. Nevertheless, this species produces floral nectar. This is surprising since orchids commonly use nectar to entice their pollinators. The pollination biology, including the chemical composition of the nectar of allogamous *Epipactis* species, is well known [6, 16, 21, 31–34], however, the composition of chemical pollinator attractants in nectar produced by self-pollinating species has not been studied so far.

In this study, for the first time, we characterize the composition of secondary compounds, including insects' attractants in the nectar produced by the autogamous *E. albensis*, and we try to explain why this taxon produces these chemicals and food for potential visiting insects while, hypothetically, it is not pollinated by them.

## Results

### Field observations

**Fig. 1:**
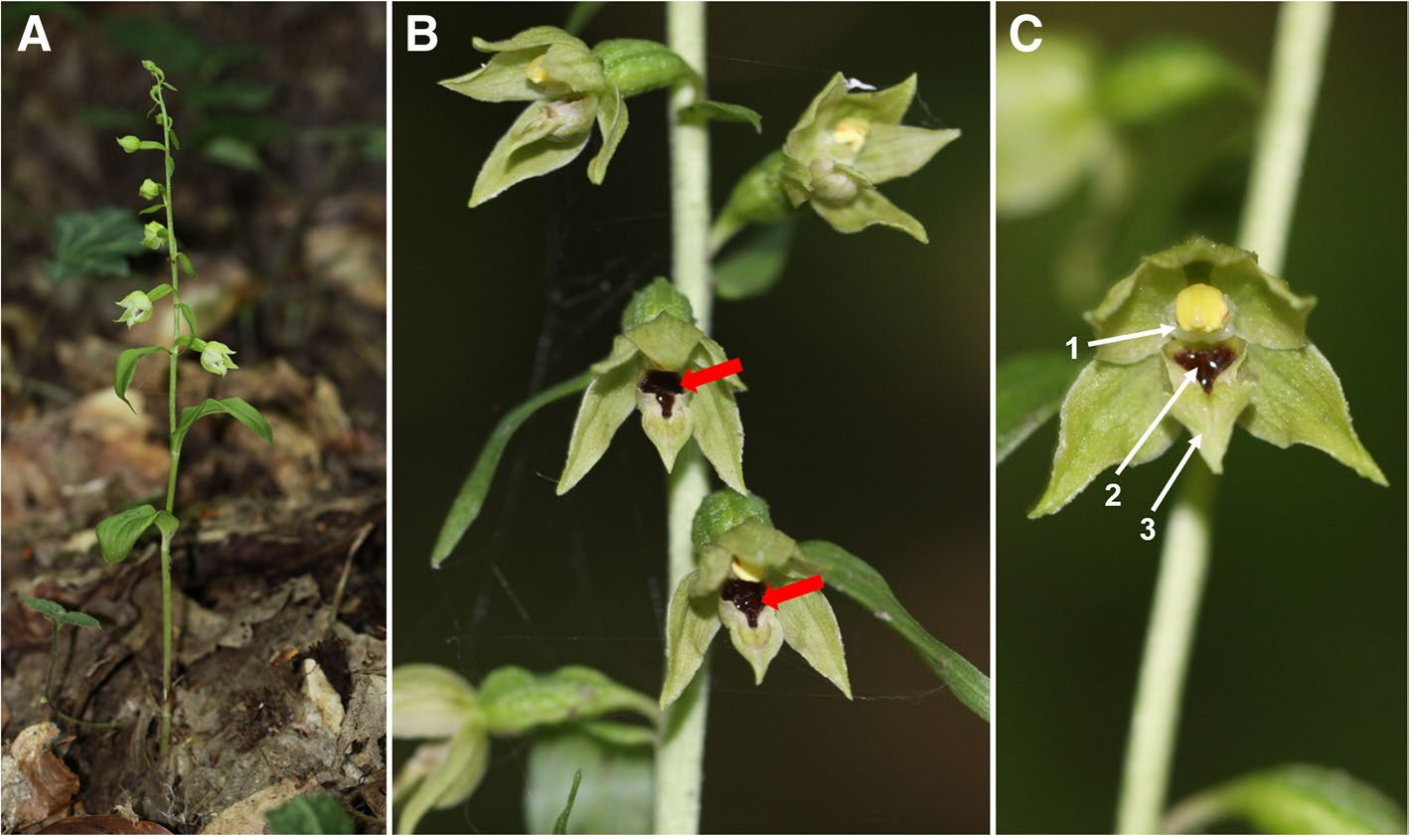
An obligate autogamous species *Epipactis albensis*. (**A**) a habit of plant; (**B**) details of flower secreted nectar. Hypochile (basal part of a labellum) with nectar are marked with red arrows (**C**) *E. albensis*: parts of the flower (1) pollinium; (2) hypochile and (3) epichile, apical part of the labellum

Observations carried out in field conditions confirmed nectar secretion between 9:00 a.m. and 6:00 p.m. in some specimens of *Epipactis albensis* (Fig. 1A). We observed visible droplets of floral nectar that accumulated inside the concave basal part of the labella named hypochile (Fig. 1B, C). In all the habitats we studied in this article, we did not find any other species of orchids or other flowering plants that produce nectar or pollen, which insects feed on.

### Observation of insect visitors

**Fig. 2:**
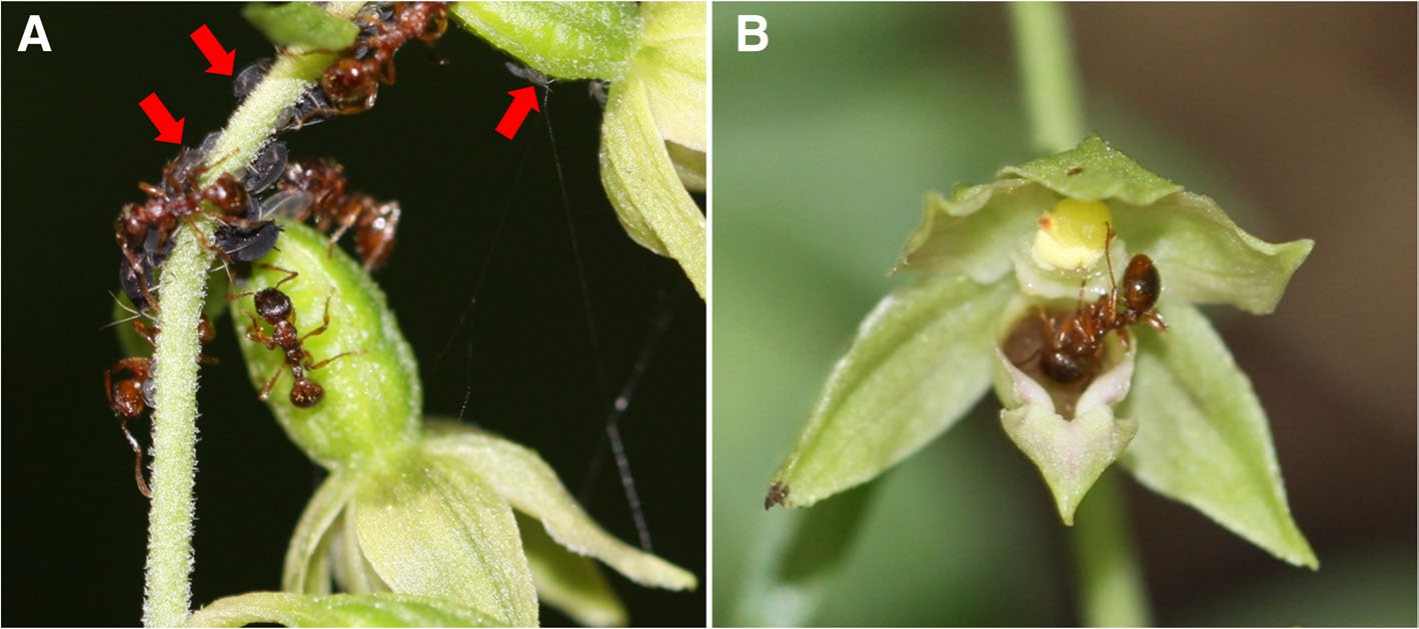
**A** Workers of red ants (*Myrmica rubra*) tending black aphids marked with red arrows. Above-ground aphid colonies producing honeydew very rich in sugar; (**B**) Visitor insects, *Myrmica rubra* (Hymenoptera) feeding nectar produced by *Epipactis albensis*, Guzice (SW Poland)

During the five-year observation in studied populations, we did not find any insects that could be considered as true pollinators of *Epipactis albensis*. Importantly, we did not observe the activity of both diurnal and nocturnal insects visiting or pollinating this orchid in the studied habitats. Sporadically we observed visitors as male and female mosquitoes (*Culex* spp.) feeding on nectar, as well as *Myrmica rubra* (Hymenoptera, Formicidae, Myrmicinae), (Fig. 2 A, B). Aphids were observed on seventeen plants (~ 4% of all plants) of *E. albensis* in all studied populations. These small insects feed on plant juices mainly from the orchids’ upper part of shoots, feeding in colonies (Fig. 2 A).

### GC–MS analyses of nectar composition

**Fig. 3:**
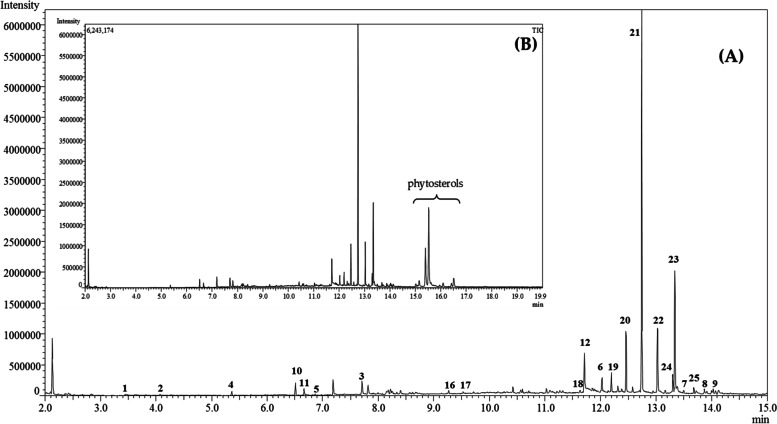
**A** Fragment and **B**, the full range of GC/MS chromatogram of *Epipactis albensis* flowers extract (dichloromethane solution). The peaks of selected compounds were numbered according to the Table 1

The dichloromethane extract of *Epipactis albensis* labella contained total of 48 compounds, most of which were identified (Table 1, Fig. 3). The extract mainly consisted of long-chain hydrocarbons and fatty acid (c.a. 74%). Among them heptacosane was the most prominent (21, Fig. 3). Only one n-alkene, i.e., 1-nonacosene (24) was identified in the sample (c.a. 2%, Table 1). Oxygen-containing compounds were less abundant accounting for only 26% of total amount of the identified organic compounds of the nectar. Among them aromatic alcohols namely monosubstituted phenols, e.g., 4-ethylphenol and derivatives of benzyl alcohols such as vanillyl alcohol (15) and metoxyeugenol (16) were identified in greater amounts than the aliphatic ones (8% vs 1.6%). The extract also contained unbranched and branched aliphatic saturated aldehydes C5-C28 (c.a. 12%) as well as aromatic ones (c.a. 4%) e.g., hyacinthin (10). Detailed analysis of the spectrum indicated also various plant sterols, e.g., campesterol, stigmasterol, gamma-sitosterol etc. among discussed compounds (Fig. 3B). The composition of the nectar of *E. albensis*, as compared to the insect-pollinated species of the genus, is poorer especially in long-chain carboxylic acids and its esters (Table 2).

**Table 1:** List of organic compounds identified in the nectar of *Epipactis albensis*

	**Compound**	**CAS Number**		**Relative amount (%)**^**a)**^
**Oxygen-containing compounds**				
1	methyl isobutyl ketone^b)^ (4-metyl-pentanone)		108–10-1	0.32
2	2-pentenal^d)^		1576–87-0	0.33
3	2,3-dimethylpentanal (2,3-dimethyl valeraldehyde)^d)^		32,749–94-3	4.01
4	heptanal^c)^		111–71-7	0.95
5	nonanal (pelargonal)^c)^		124–19-6	0.36
6	Octadecanal^c)^		638–66-4	3.69
7	heptacosanal^c)^		72,934–03-3	0.98
8	octacosanal^c)^		22,725–64-0	1.47
9	octacosanol^c)^		557–61-9	1.55
10	phenylacetaldehyde (hyacinthin)^c)^		122–78-1	2.69
11	4-methylphenol (*p*-cresol)^b)^		106–44-5	0.83
12	4-ethylphenol^c)^		123–07-9	5.34
13	4-hydroxybenzaldehyde^b)^		123–08-0	1.27
14	4-(hydroxymethyl)phenol^c)^		623–05-2	0.42
15	4-hydroxy-3-methoxybenzyl alcohol^b)^ (vanillyl alcohol)		498–00-0	0.76
16	4-allyl-2-(methoxymethoxy)-phenol^c)^ (methoxyeugenol)		6627–88-9	0.63
17	3,5-dimethoxy-4-hydroxybenzaldehyde (syringaldehyde)^d)^		134–96-3	0.40
**Long-chain hydrocarbons and fatty acids**^b)^				
18	tricosane		638–67-5	0.40
19	pentacosane		629–99-2	1.58
20	hexacosane		630–01-3	6.17
21	heptacosane		593–49-7	39.73
22	octacosane		630–02-4	7.83
23	nonacosane		630–03-5	13.57
24	1-nonacosene		18,835–35-3	2.03
25	triacontane		638–68-6	0.80
26	palmitic acid (hexadecanoic acid)		57–10-3	1.86
**Unidentified compounds**				14 substances

^**a)**^ with respect to the MS response factors

^**b)**^ identified by comparison to standards

^c)^ NIST Quality Score of ≥ 90

^d)^ NIST Quality Score of ≥ 84

**Table 2:** Selected chemical compounds, known as insect attractants, identified in the nectar of autogamous *Epipactis albensis* and closely related allogamous species *Epipactis helleborine*. * Data based on [6, 21, 32, 33]

Chemical compound	*Epipactis albensis*	Epipactis helleborine*
benzoic acid	-	+
benzylalcohol	-	+
pentadecanol	-	+
heptadecanol	-	+
eicosanol	-	+
2-pentenal	+	+
heptanal	+	+
nonanal	+	+
hexadecanal	-	+
octadecanal	+	+
nonadecanal	-	+
4-hydroxybenzaldehyde	+	+
phenylacetaldehyde	+	+
4-hydroxy-3-methoxy-benzaldehyde (vanillin)	-	+
4-hydroxy-3-methoxy-benzylalcohol (vanillyl alcohol)	+	+
2-metoxy-4-(2-propenyl)-phenol (eugenol)	-	+
2,6-dimethoxy-4-(2-propenyl)-phenol (methoxyeugenol)	+	+
4-methylphenol	+	+
2,6-dimethoxy-phenol (syringol)	-	+
3,5-dimethoxy-4-hydroxybenzaldehyde (syringaldehyde)	+	-
4-(hydroxymethyl)phenol	+	+
eicosane	-	+
heneicosane	-	+
tricosane	+	+
pentacosane	+	+
hexacosane	+	+
heptacosane	+	+
octacosane	+	+
heneicosane	+	+
hexadecanoic acid	+	-
tetraeicosanoic acid	-	+
oleic acid	-	+
octadecenoic acid	-	+
pentadecenoic acid	-	+
heptadecenoic acid	-	+
hexadecenoic acid	-	+
eicosanoic acid methyl ester	-	+
tetracosanoic acid methyl ester	-	+
pentadecenoic acid methyl ester	-	+
hexadecenoic acid methyl ester	-	+

## Discussion

The evolutionary shift from outcrossing to self-fertilization is one of the most frequent evolutionary transitions in plants [35]. As early as the nineteenth century, Darwin argued that outcrossed progeny of plants is usually more vigorous than those produced by self-fertilization [36, 37]. This observation led him to interpret many features of flowering plants as adaptations for outcrossing [38]. It is now believed that about 10–15% of flowering plants are predominantly self-fertilizing [35]. In flowering plants, autogamy has the disadvantage of producing low genetic diversity in the species that use it as the predominant mode of reproduction [39].

Self-pollination has also been found in other orchid genera, including *Cephalanthera* and *Epipactis* [40]. Autogamous species of the *Epipactis* genus arose secondarily as a result of adaptation to colonization of poor habitats in terms of the presence of potential pollinators. From a pollinator's perspective, a flower provides food, typically in the form of nectar and pollen. The production of nectar by the self-pollinating *E. albensis*, with a chemical composition similar to the nectar of closely related allogamous species *E. helleborine*, confirms that autogamy is a secondary process that is the adaptation of plants to the absence of pollinating insects in their habitats. This is exactly the situation we have observed in three surveyed populations. It is worth noting that in none of the populations we studied, we found no other associated species that produce nectar-rich or pollen-rich flowers. In dark habitats, environments poor in nectar-rich species of herbaceous plants, insects will not find food, which explains the lack of observation of flower visits. The studied plants do not produce the viscidium, which distinguishes them from allogamic *Epipactis* taxa, but they have not yet blocked the attractant synthesis pathway, which may suggest that *E. albensis* is a relatively young species in its evolutionary history. This is also confirmed by phylogenetic research [15].

The chemical composition of *E. albensis* nectar confirms the assumption that autogamy in *Epipactis* is a secondary process. Thus, the differences in gynostemium structure that we observe in self-pollinating taxa evolved earlier than nectar synthesis blockade.

Our research has shown that *E. albensis* is also a species with very interesting relationships with visiting insects. We observed, but rare, the male and female of *Culex* spp. mosquitoes feeding the nectar produced by *E. albensis* flowers, which is confirmed by the literature data according to which these insects feed on nectar, aphid honeydew, and plant juices [41, 42]. Interestingly, in the analyzed flower extract we identified several compounds that attract *Aedes* spp. mosquitoes [42] such as heptanal, nonanal, phenylacetaldehyde (hiacintin) and eugenol derivatives, which may explain the interest of these insects in flowers of *E. albensis*. Admittedly, mosquitoes are generally considered nectar thieves that consume nectar rather than effective pollinators of plants. There are published scarce data showing conclusive evidence for pollination of *Tanacetum vulgare* by *Culex pipiens*, and role of several other mosquito species in potential pollination of other species of the Asteraceae family, i.e. *Achillea millefolium*, *Leucanthemum vulgare*, *Solidago canadensis* [43] The association between the *Platanthera* and *Aedes* spp. mosquitoes is one of the few examples that shows mosquitoes as effective pollinators of orchids [42, 44]. Unfortunately, our research results do not confirm that mosquitoes could be effective pollinators of *Epipactis albensis*. Firstly, because the observed male and female of *Culex* spp. are too small to transfer *E. albensis* pollinia, and secondly because the structure of *E. albensis* flowers prevents effective pollination by insects. This species of orchid does not produce the viscidium, a special structure for attachment of the pollinia to the pollinators.

Representatives of Aphididae (Fig. 2), observed by us on flowers of *E. albensis* are not pollinators but they feed on vegetative shoots and flowers of orchids sucking plant juices. Insects from this group, e.g., *Aphis ilicis* have been previously observed on *E. helleborine* [31] and *Epipactis atrorubens* [33]. Similarly, we have not observed the transfer of pollen by *Myrmica* ants visiting *E albensis*; instead, these insects readily collect honeydew from black aphids and feed on flower nectar [33].

Mutualistic relationships between ants and aphids are well studied, aphids provide ants with sugar-rich honeydew as a source of food and the ants protect the aphids against various natural enemies and improve the hygiene of the aphid colony [45].

In general, insect-pollinated plants communicate with their pollinators through a number of floral signals, such as unique flower shape, coloration and odour [46]. Floral scents are among the key signals in many plant-pollinator systems for attracting pollinators from both short and long distances [46]. If a plant is not pollinated by insects, as *E. albensis* we studied, why it is producing strong chemical attractants? It is possible, and it is an evolutionary legacy of ancestors. There is a noticeable similarity in the scent bouquets of the *E. albensis* and the *E. helleborine* studied earlier [6, 21, 32, 33] (Fig. 2 and Table 2). The likeness of the scent profiles of both species certainly confirms that two taxa are closely related. It should be noted, however, that the list of semiochemicals identified in the investigated nectar is much poorer compared to *E. helleborine* (Table 1). This might be explained that in contrast to studied here *E. albensis*, *E. helleborine* is well-known as insect-pollinated plant [6, 16, 31, 33]. Our previous studies have shown that the main pollinators of *E. helleborine* include representative of Hymenoptera, e.g. *Bombus* sp., *Apis mellifera*, *Vespula vulgaris* and *V. germanica* and well as Diptera, i.e., *Episyrphus balteatus* [16, 31, 33].

In the nectar sample of *E. albensis* we mainly identified various long-chain alkanes and only one fatty acid (Table 1 and Fig. 3). As for long-chain alkanes, their presence in the sample is not surprising since these types of compounds are ubiquitous in the wax layer of the flower of various plants [47]. In *E. albensis* flowers, the inner part of the lip (hypochil) in which the nectar is produced has such a waxy layer. Similar alkanes were identified in the nectar we compared (Table 2). Some of them, i.e., heneicosane and hexacosane were identified as Hymenoptera attractants, substances luring bumblebees (*Bombus* sp., Hymenoptera, Apidae). Interestingly, heneicosane is used as a pheromone by the queen or king termites in the species *Reticulitermes flavipes* [48]. These hydrocarbons (heneicosane and hexacosane) have also been found in *Serapias*, another genus of orchids [49, 50]. However, in contrast to the high number of fatty acids and its esters identified in the *E. helleborine* lack of these compounds in *E. albensis* could support the absence of pollinating insects. It is well known, that saturated and unsaturated fatty acids are strongly associated with the biosynthesis of alkenes with different double-bond position, the key components of pollinators’ sex pheromones [51].

Surprisingly, we found in flower extract of *E. albensis* long-chain aliphatic saturated aldehydes described in the literature as insect’s attractants, e.g., nonanal and octadecanal (Table 1). Peach-specific aldehyde nonanal (pelargonal) is considered an insect attractant or pheromone. This semiochemical attracts numerous species of butterflies, e.g., female oriental fruit moths, *Grapholita molesta* (Lepidoptera: Tortricidae) [52]. This compound is also attractive to the Africanised honey bee *Apis mellifera scutellate* (Hymenoptera, Apidae), as well as it lures species from different insects’ groups, both the ant subfamilies Formicinae and Myrmicinae (the order Formicidae) [53]. It is therefore possible that the activity of *Myrmica rubra* on *E. albensis* we observed was due to the insect’s reaction to this compound and/or results of synergistic effect of other chemicals. Octadecanal is another example of semiochemicals that attracts different species of ants, e.g., *Apterostigma pilosum* (Myrmicinae, Attini) [54] *Polyergus rufescens* (Formicinae, Formicini) [55], or *Cerapachys jacobsoni* (Cerapachyinae, Cerapachyini) [56]. This chemical compound is also a potential attractant for, e.g., *Psithyrus vestalis* (Apinae, Bombini) [57] and *Hypotrigona ruspolii* (Meliponinae, Meliponini) [58]. Interestingly, the representatives of Lepidoptera also react to it, e.g., *Andraca bipunctata* (Bombycidae, Oberthuerinae) [59], *Earias vittella* (Noctuidae, Chloephorinae [60], *Cerconota anonella* (Oecophoridae, Stenomatinae) [61], *Heliconius melpomene plesseni* (Nymphalidae, Heliconiinae, Heliconiini) [62], or *Manduca sexta* (Sphingidae, Sphinginae, Sphingini) [63].

Another important compound identified in the nectar of *E. albensis* is 2-pentenal. We previously identified both this compound and its derivatives, i.e., 2-pentanol in *E. helleborine* (Jakubska-Busse, unpublished data). This semiochemical is attractive to, among others, jewel bugs *Chrysocoris stolli* (Heteroptera, Scutelleridae, Scutellerinae) [64] and the scuttle fly genus *Megaselia* (Diptera, Phoridae, Metopiinae, Metopinini) [65].

*E. albensis* also produces phenylacetaldehyde, an aroma organic compound known as hyacinthin or benzeneacetaldehyde. This semiochemical was identified in the nectar of *E. helleborine* and is emitted by other orchids, such as moth-pollinated *Gymnadenia odoratissima* [66]. The aroma of a pure compound can be characterized as honey-like, sweet, rose, green and grassy and it is widely used in perfumery as fragrance to impart hyacinth, daffodil, or rose nuances [67]. It is notable for being a floral attractant for numerous species of Lepidoptera from e.g., the orders Noctuidae, Sphingidae, Geometridae, Danaidae, Crambidae or Pyralidae [53, 68]. Hyacinthin is also an attractant of other insect groups, including e.g., the western honey bee *Apis mellifera* (Hymenoptera, Apidae) [69] or march fly *Plecia nearctica* (Diptera, Bibionidae) [70]. Although the plants of *E. albensis* produce hyacinthin, we were unable to observe any insects visiting the flowers, even those active at night. Interestingly, this compound is also attractive to the Formicidae (Hymenoptera), whatmay explain our observation of ants visiting flowers of *E. albensis* [53].

Other compounds worth mentioning are vanillin and syringaldehyde, well-known attractants for many different groups of insects, e.g., different species of bark beetles of the genus *Scolytus* (Coleoptera, Scolytidae), e.g., *Scolytus multistriatus* [71], as well as for leaf-footed bugs *Leptoglossus phyllopus* (Heteroptera, Coreidae) [72] and for neotropical butterfly *Heliconius melpomene rosina* (Lepidoptera, Nymphalidae) [73]. The presence of numerous vanillin and eugenol derivatives in flower nectar was previously found in other insect-pollinated *Epipactis* species [6, 21, 32, 33]. Eugenol is a widespread and important scent compound, which has also been identified in floral scent emitted by another orchid, i.e., *Cypripedium calceolus* [74] and *Gymnadenia* species [46]. Although we could not identify/detect vanillin in the nectar of *E. albensis*, the presence of vanillyl alcohol and 4-hydroxybenzaldehyde, compounds considered as intermediates of the vanillin biosynthetic pathway [75], might confirm the ability of this species to synthesize vanillin.

It is worth emphasizing that the nectar and scent chemical composition of other *Epipactis* species we have previously studied, i.e., *E. atrorubens*, *E. purpurata* and *E. palustris*, is different from *E. helleborine* and *E. albenis* [6, 21, 33], which confirms the distinctiveness of these species.

Identified in *E. albensis* plant sterols, e.g., campesterol, stigmasterol, gamma-sitosterol are not attractants and are irrelevant in the pollination biology of *Epipactis* species. Phytosterols are a group of naturally occurring compounds found in plant cell membranes that can also modulate the activity of membrane-bound enzymes [76]. These compounds are also linked to plant adaptation to temperature and plant immunity against pathogens [77]. However due to their low importance as insect attractants their occurrence in the flowers has not been discussed. In the sample trans-phytol (*E*-3,7,11,15-tetramethyl-2-hexadecen-1-ol), a diterpene alcohol obtained from the degradation of chlorophyll was also identified.

## Conclusions

Our research points to the possibility that lack of pollinators in habitats can stimulate a transition to autonomous selfing as reproductive assurance [78, 79]. The production of numerous chemical attractants by self-pollinated *E. albensis* confirms the evolutionary transition from ancestral insect-pollinating species to obligatory autogamous. From an evolutionary perspective, it is likely that the next step in the evolution of *E. albensis* will be to gradually block the synthesis of substances luring insects, since the production of insect attractants, which are not crucial for pollination biology of this orchid, is redundant. There is also the possibility that the nectar does not disappear since the attraction of insects such as ants might provide a protective adaptation against herbivore damage.

## Methods

### Plant materials

Fragments of ca. 300 fresh flowers with visible nectar secretion (hypochile) of *Epipactis albensis* used for the chemical analyses, were collected from three natural populations, i.e., 30 individuals of the populations located in the vicinity of Guzice near Polkowice, ca. 30 individuals from Wałkowa near Milicz (Lower Silesia, SW Poland), and from 32 individuals from Siechnice near Wrocław, between 24 July 2017 and 14 August 2021. AJB undertook the formal identification of the plant material used in our study. Voucher specimens without a collection number (*sine numero*) representative of all samples are stored at the Herbarium of Department of Botany, University of Wroclaw, Poland. Because *E. albensis* is a legally protected species in Poland and publishing its natural population coordinates of Global Positioning System (GPS) is an inappropriate protective procedure, we do not provide the exact location of the research sites. GPS coordinates are available from the authors upon request. Field observations and material sampling done with permission nos. WPN.6205.134.2017.IL, WPN.6400.33.2018.IL, WNP.6400.29.2019.AR, WNP.6205.87.2019.AR, WPN.6400.24.2020.MH, WPN.6205.113.2021.MR, and WPN.6400.20.2021.MR from the Regional Directors for Environmental Protection.

### Field observations of insects’ activity

The observations were conducted during the peak of the plant flowering period from 24 July to 17 August 2017–2020 and from 29 July to 13 August 2021 in the above-mentioned populations, located in Lower Silesia (SW Poland). The observations were carried out independently using two methods, i.e., four remotely controlled cameras and direct observations were made over a span of 2–6 h per plant or the group of plants, covering daylight hours (9:00 a.m.—6:00 p.m.). The visitors' insects were photographed/documented using a Nikon D50 camera with a Tamron 90 mm f/2.8 SP Di Macro lens, captured in the field conditions by AJ-B and identified by specialists. We used four digital video cameras Sony HDR-CX450, equipped with a set of SD cards and batteries for both daytime and nighttime recordings. In total, 728 h of directed observations were conducted.

### GC–MS analyses of nectar composition

Prepared samples of the basal part of the *E. albensis* labella (hypochile) containing nectar (0.22 g) were collected into the 2 mL glass vial followed by adding of 0.5 mL of dichloromethane (Sigma-Aldrich, 99.9%) at room temperature. The dichloromethane was used to extract foliar nectar drop. All samples for analysis were collected on the same day. The extract was stored at -20ºC until used for GC–MS analyses. GC–MS chromatography was performed on GCMS-QP2010SE SHIMADZU equipped with a mass selective detector (MS scan 17–550 m/z) and Zebron ZB-5 ms (30 m 0,25 mm; Phenomenex) column operated at 40ºC for 3 min, followed by heating to 320 °C at rate of 30 °C/min and then at 320 °C for 7.7 min. Helium was used as a carrier gas.

Identification of the compounds was carried out using the NIST17 database. In addition, compounds: methyl isobutyl ketone, octanal, 4-hydroxybenzaldehyde, *p*-cresol, vanillyl alcohol and palmitic acid were identified by comparison with standards.

**Fig. 4:**
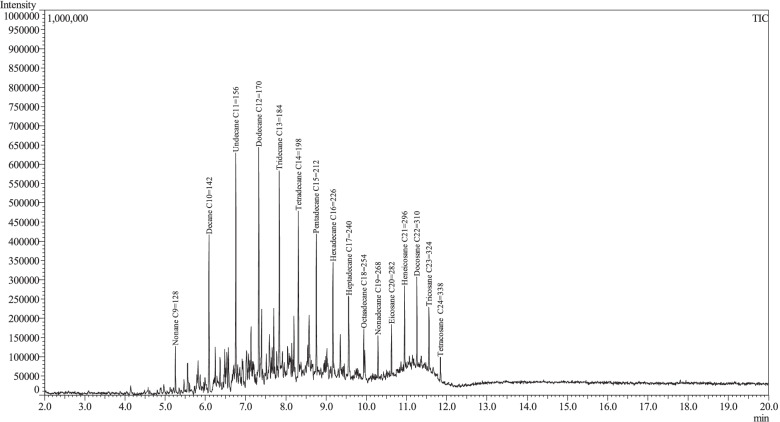
GC–MS chromatogram of sample containing C_9_-C_24_ hydrocarbons

**Fig. 5:**
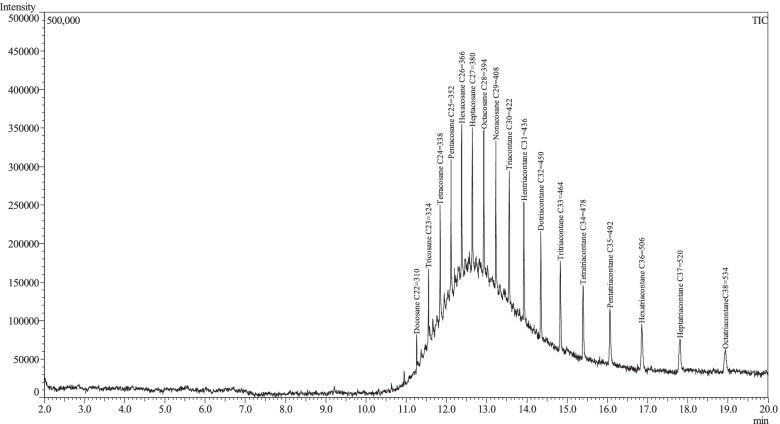
GC–MS chromatogram of sample containing C_22_-C_38_ hydrocarbons

For identification of long-chain hydrocarbons, samples of C9-C24 and C22-C38 alkanes were analyzed by GC–MS using the same oven and column parameters and their spectra were compared with those obtained in the extract (Figs. 4–5). Relative amounts of compounds (%) in the sample were calculated from the MS detector response. In order to determine response factors for compounds in the investigated extract, the reference sample containing standards: methyl isobutyl ketone, octanal, 4-hydroxybenzaldehyde, *p*-cresol, vanillyl alcohol, palmitic acid and hexadecane was analyzed by GC/MS using the same parameters as in the case of the investigated sample. The relative amounts (%) of aliphatic aldehydes and long-chain hydrocarbons were estimated based on a comparison with octanal and hexadecane, respectively. The relative amount of 4-ethyl-phenol was estimated based on a comparison with *p*-cresol. In the case of 4-(hydroxymethyl)phenol, methoxyeugenol and syringaldehyde vanillyl alcohol was used for estimation of their relative amounts.

## Data Availability

All data generated or analysed during this study are included in this published article.

## Abbreviation

GC-MS: Gas chromatography-mass spectrometer
